# Machine learning framework for gut microbiome biomarkers discovery and modulation analysis in large-scale obese population

**DOI:** 10.1186/s12864-022-09087-2

**Published:** 2022-12-23

**Authors:** Yaoliang Liu, Jinlin Zhu, Hongchao Wang, Wenwei Lu, Yuan Kun LEE, Jianxin Zhao, Hao Zhang

**Affiliations:** 1 State Key Laboratory of Food Science and Technology, Jiangnan University, P. R, Wuxi, 214122 Jiangsu China; 2 School of Food Science and Technology, Jiangnan University, Wuxi, 214122 Jiangsu China; 3 Department of Microbiology & Immunology, Yong Loo Lin School of Medicine, National University of Singapore, 21 Lower Kent Ridge Rd, Singapore, Singapore; 4 International Joint Research Laboratory for Pharmabiotics & Antibiotic Resistance, Jiangnan University, Wuxi, China; 5 National Engineering Research Center for Functional Food, Jiangnan University, Wuxi, 214122 Jiangsu China; 6 Wuxi Translational Medicine Research Center and Jiangsu Translational Medicine Research Institute Wuxi Branch, Wuxi, 214122 Jiangsu China

**Keywords:** Obesity, Gut microbiome, Machine learning, Geography, Ensemble learning, Counterfactual explanation, Reinforcement learning

## Abstract

**Background:**

The gut microbiome has proven to be an important factor affecting obesity; however, it remains a challenge to identify consistent biomarkers across geographic locations and perform precisely targeted modulation for obese individuals.

**Results:**

This study proposed a systematic machine learning framework and applied it to 870 human stool metagenomes across five countries to obtain comprehensive regional shared biomarkers and conduct a personalized modulation analysis. In our pipeline, a heterogeneous ensemble feature selection diagram is first developed to determine an optimal subset of biomarkers through the aggregation of multiple techniques. Subsequently, a deep reinforcement learning method was established to alter the targeted composition to the desired healthy target. In this manner, we can realize personalized modulation by counterfactual inference. Consequently, a total of 42 species were identified as regional shared biomarkers, and they showed good performance in distinguishing obese people from the healthy group (area under curve (AUC) =0.85) when demonstrated on validation datasets. In addition, by pooling all counterfactual explanations, we found that *Akkermansia muciniphila*, *Faecalibacterium prausnitzii, Prevotella copri, Bacteroides dorei, Bacteroides eggerthii, Alistipes finegoldii, Alistipes shahii, Eubacterium* sp. *_CAG_180,* and *Roseburia hominis* may be potential broad-spectrum targets with consistent modulation in the multi-regional obese population.

**Conclusions:**

This article shows that based on our proposed machine-learning framework, we can obtain more comprehensive and accurate biomarkers and provide modulation analysis for the obese population. Moreover, our machine-learning framework will also be very useful for other researchers to further obtain biomarkers and perform counterfactual modulation analysis in different diseases.

## Background

Over the past 40 years, the number of obese people has increased rapidly worldwide [1]. Several studies indicate that the global obesity prevalence will exceed 20% for men and 18% for women by 2025 [2]. Obesity is linked to multiple pathologies, such as cardiovascular disease, diabetes, cancer, and depression [3, 4]. It has been reported that the combination of unhealthy dietary patterns, environmental factors, insufficient physical activity, and genetics may be the major cause of obesity [5, 6]. Other factors, including stress [7], sleep duration [8], poor calcium intake [9], and the gut microbiome [10, 11] have been proposed as key players in the development of obesity.

There is growing interest in the role of the gut microbiome in regulating obesity [11]. Recent studies have shown that the gut microbiome affects obesity by influencing energy balance, gut barrier function, inflammation, and the production of significant bioactive factors, including short-chain fatty acids (SCFAs), lipopolysaccharides, and bile acids [12, 13]. The core biomarkers of obesity have been inconsistently reported across individual studies [14], possibly owing to the complexity of host background factors and the inconsistency of analytical approaches. Among these host background factors, geographical differentiation is regarded as the most important [15]. However, from the perspective of methodology, current studies lack a systematic way to identify biomarkers, which eventually leads to an incomplete and inconsistent understanding of the gut microbiome biomarkers of obese people. At the same time, when analyzing the modulation of the gut microbiome, most studies simply compare the difference in gut microbiome abundance between obese people and healthy people [16], which ignores many biomarkers that may play an important role in machine learning. Moreover, there is still a challenge in personalized regulation of the minimum subset of the targeted gut microbiome to restore the health status of obese individuals, not to mention finding broad-spectrum targets with consistent modulation across different geographic groups.

Thus, there is a need to build and validate a relationship between the gut microbiome and obesity across a wide range of populations in different regions using a comprehensive and systematic approach. Generally, collecting many microbiome samples from different regions requires considerable time and effort, and only large research centers can conduct them. However, with recent progress in the call for data-sharing policies [17], an increasing number of shotgun metagenomic sequencing datasets are accessible from published studies worldwide. By pooling these datasets, it is possible to study obesity-associated shared biomarkers on a large scale.

This study introduced a machine learning framework to obtain regional shared biomarkers and perform modulation analysis with 870 raw shotgun stool metagenomes representing five countries from published studies. This machine-learning framework can be divided into two parts. First, a heterogeneous ensemble feature selection method, including filter, embedded, and wrapper, was established to obtain a comprehensive biomarker subset from each country. Notably, to remove geographical factors, we consider the intersection of each country as a regional shared biomarker. Each of the selected biomarkers plays a crucial role in obesity, regardless of regional background. Biomarkers were evaluated using an additional validation cohort to confirm the generalization of predictive capability. Second, a deep reinforcement learning model was developed to create counterfactual instances for the gut microbiome of obese individuals. A counterfactual instance states the minimum necessary changes to some biomarkers of a given obese individual that result in a targeted prediction of the machine learning model, whereas the targeted prediction is healthy in this work. The counterfactual instance is intuitive because it tells each obese individual what to do to achieve the desired outcome by altering the minimum necessary biomarkers. In doing so, we can provide a precise modulation strategy for each obese individual from the perspective of counterfactual machine learning. In summary, this study 1) comprehensively obtained regional shared biomarkers related to obesity, 2) performed counterfactual modulation analysis for individual people, and 3) inferred a set of broad-spectrum consistent targets for cross-regional obesity populations.

## Results

### Integration of human metagenomic data from different countries

**Fig. 1:**
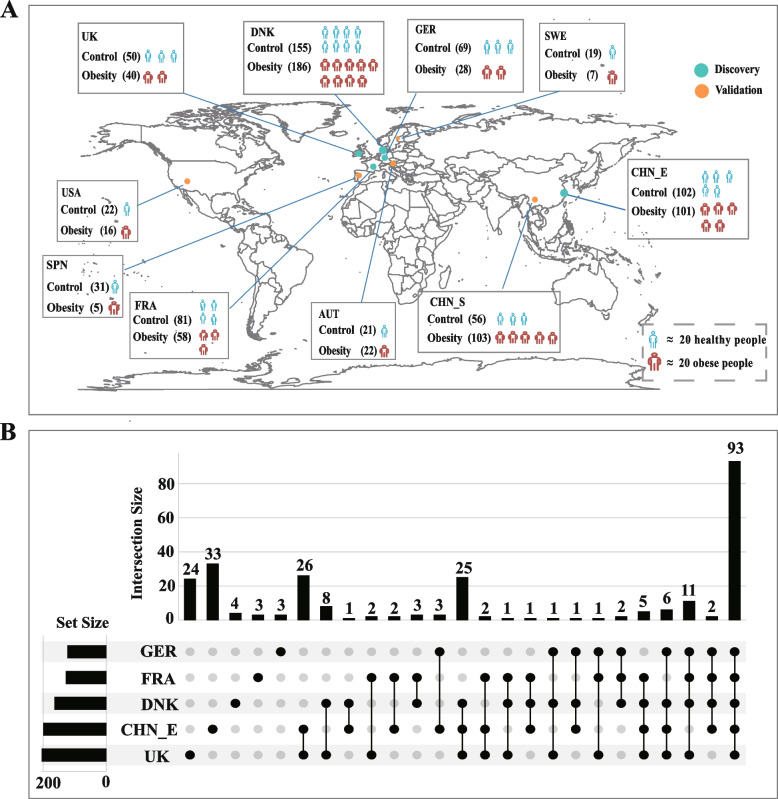
Overview of the obese and healthy population included in this study**.** **A** Global map representing 1172 samples with shotgun metagenomic data, including 606 obese individuals and 566 control individuals. The discovery populations included Denmark (DNK), Germany (GER), France (FRA), the United Kingdom (UK), and China Eastern (CHN_E). The rest of the countries formed the validation dataset, including the United States (USA), Austria (AUT), Sweden (SWE), Spain (SPN), and China Southern (CHN_S). **B** The UpSet plot reveals the number of species (excluding low prevalence species) in each discovery cohort and shared by combinations of these datasets. The set size represents the number of species in each country/region. The connected dots mean the common species across connected countries/regions and the number on each column represents the amounts of species

To exclude the influence of geographical factors on the biomarkers of body mass index (BMI), we need to obtain many samples from different regions. Therefore, we retrieved metagenomic datasets from studies related to obesity and combined the datasets of the control group from various studies on other diseases (see Methods). We collected 1172 raw shotgun stool metagenomes from published studies (Fig. 1A). Among these samples, the discovery cohort consisted of 870 individuals, including 414 obese individuals and 456 healthy individuals across five countries (Denmark, Germany, France, the United Kingdom, and Eastern China). The validation cohort consisted of 149 obese individuals and 153 healthy individuals from other countries and regions (Fig. 1A).

After downloading raw metagenomes, we first reprocessed them uniformly to remove a major nonbiological source of variance between different studies [18]. The species abundance of each individual was determined using the MetaPhlAn2 pipeline (see Methods). To obtain sufficient and precise taxonomic annotations, our study mainly focused on species-level information. Therefore, an aggregate of 701 species was obtained from all samples. After removing the low-prevalence species (the prevalence rate in the obese or healthy population in any country is not more than 10%), 264 species remained. Among them, 93 species were found in every country (Fig. 1 B), which formed the basis for the further discovery of regional shared biomarkers in obesity.

### Gut microbiome composition can be affected by BMI and geographic factors

**Fig. 2:**
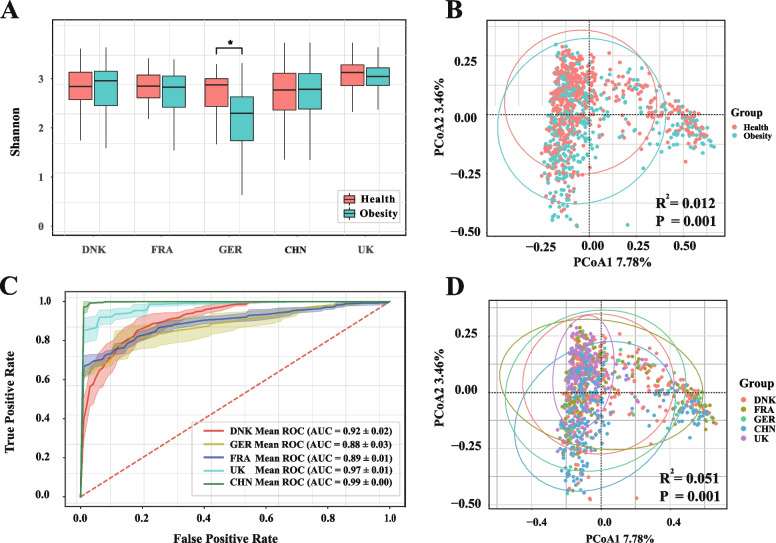
The influence of geography and BMI factors on the composition of the gut microbiome. **A** The box plots (box limits, upper and lower quartiles; center line, median; whiskers, 1.5 × interquartile range) show the alpha diversity assessed by the Shannon index of healthy and obese groups in five countries. Only in GER, the Shannon index of healthy people is significantly higher than that of obese people. (Wilcoxon rank-sum test, *P* < 0.05). **B** Principal coordinates analysis (PCoA) plot dependent on Bray–Curtis distances shows that obese (green) and healthy (red) people have essentially different gut microbiome profiles as PERMANOVA (*R*^*2*^ *=* 0.012*, P =* 0.001). Each point in the plot corresponds to one person. The ellipses correspond to the 95% confidence region. **C** The receiver operating characteristic (ROC) plots shows the classification effect of geographical factors on different countries by the Random Forest (RF) model. **D** The identical PCoA plot shows that the gut microbiome composition of people in different regions is significantly different (*R*^*2*^ = 0.051, *P* = 0.001). Each color in the plot corresponds to one country. Similarly, each point in the plot corresponds to one person

In this study, we first evaluated the impact of BMI on the gut microbiome composition. Compared with healthy individuals, obese individuals had lower alpha diversity in general, particularly for GER (Wilcoxon rank-sum test, *P* < 0.05) (Fig. 2A), which was consistent with most previous studies [19]. Moreover, by employing principal coordinate analysis (PCoA), we also found significant differences between the obese and healthy groups (permutational multivariate analysis of variance (PERMANOVA), *R*^*2*^ = 0.012, *P* = 0.001) (Fig. 2B).

We examined the influence of geographical factors on the gut microbiome. First, we assessed whether geographical factors could differentiate samples from different countries using machine learning models. After 10 times 5-fold cross-validation for each country’s cohort and the corresponding remaining cohorts, we found that the geographical factors, with the Random Forest (RF) machine learning model, showed high performance in distinguishing individuals from different countries (mean AUC = 0.93) (Fig. 2C). In addition, the distinction between China and other countries was the highest (AUC = 0.99) (Fig. 2C), which may be attributed to the fact that China is the farthest away from other countries. In contrast, when using PCoA ordination, we also found significant differences between groups in different countries/regions (permutational multivariate analysis of variance (PERMANOVA), *R*^*2*^ = 0.051, *P* = 0.001) (Fig. 2D), which further demonstrated the influence of geographical factors on the gut microbiome. In conclusion, the gut microbiome composition of people is influenced by BMI and geographical factors.

### Identification of regional-shared biomarkers through ensemble feature selection

**Fig. 3:**
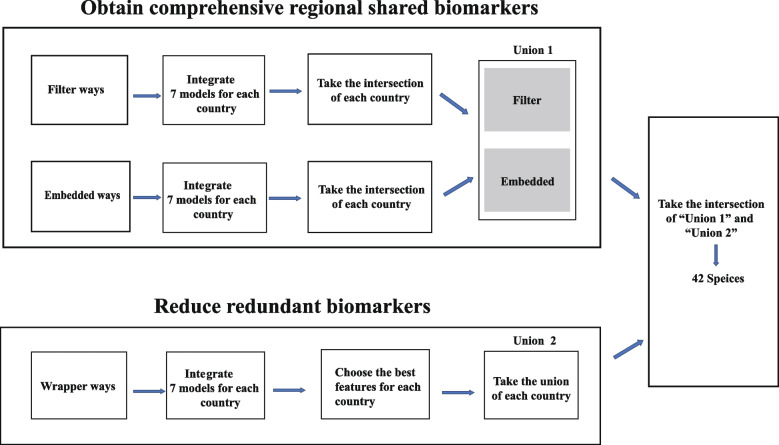
The flow chart for identification of regional shared biomarkers in the obese population

Considering the high-dimensional, stochastic [20], sparse, and heterogeneous [21] nature of microbiome datasets, we developed an ensemble feature selection diagram by integrating filter, embedded, and wrapper methods to obtain more comprehensive obesity biomarkers from multiple perspectives, which, in turn, can avoid the bias caused by a single feature selection method. The overall flowchart can be divided into two parts: obtaining comprehensive regionally shared biomarkers and removing redundant biomarkers (Fig. 3).

**Fig. 4:**
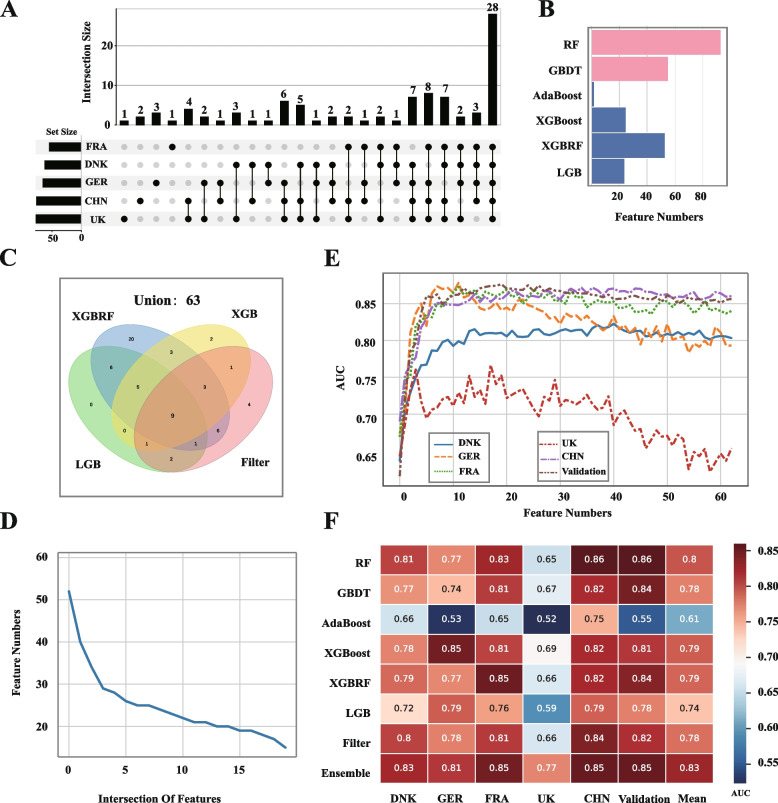
Identical regional shared biomarkers of obesity. **A** UpSet plot revealing the amount of filter common species in each discovery cohort and shared by combinations of these datasets. The set size represents the number of biomarkers in each country. The connected dots mean the common biomarkers across connected countries and the number on each column represents the amounts of biomarkers. **B** The bar plot shows the number of regional shared biomarkers obtained by different methods. The pink color represents the unstable methods. **C** The Venn plot shows the union of different feature selection methods. **D** The line plot shows that the results of the Gradient boosted regression trees (GBDT) model have poor stability. The blue line corresponds to the intersection of the result, with the increase in repetition times. **E** The line plot shows that the AUC of 5 countries with the increase of the regional shared feature numbers by the XGBoost model. **F** The heatmap shows the ability of different methods to distinguish between obese and healthy individuals in each country and validation cohort

For the first part, seven classic filter methods were integrated to obtain biomarkers from each country, including Pearson’s correlation coefficient, Spearman’s correlation coefficient, Kendall’s correlation coefficient, MIC, Pearson’s partial correlation coefficient, Spearman’s partial correlation coefficient, and Kendall’s partial correlation coefficient (see Methods). Thereafter, we consider the intersection of each country to exclude geographical factors. As a result, 28 species were obtained as regionally shared biomarkers from the perspective of filter methods (Fig. 4A). Similarly, seven models were selected as representative embedded methods, including Decision Tree (DT), Random Forest (RF), Gradient boosted regression trees (GBDT), eXtreme Gradient Boosting (XGBoost, XGBRF), Adaptive Boosting (AdaBoost), and LGB (Light Gradient Boosting Machine (LGB) (see Methods). These models have been commonly used in datasets related to the gut microbiome [22–25]. However, owing to the limitations of these methods, the four models failed to identify regional shared biomarkers. Specifically, the DT model resulted in an empty feature subset for the intersection of the five discovery cohorts; conversely, the RF model was inclined to select almost all features as regional shared biomarkers and tended to be inconsistent at different times (Fig. 4B). For the GBDT, the number of regional shared features ranged from 47 to 60 at different times, and the shared biomarkers over cumulative repeated selection also gradually decreased with the increase in repeat times (Fig. 4D), which suggested that the GBDT model was also unstable when choosing regional biomarkers. Although the stability of AdaBoost can be ensured, it was still questionable to obtain regional shared biomarkers because only two species were selected (Fig. 4B), which could not stay in line with common sense. Finally, only three embedded models (XGBoost, XGBRF, and LGB) were reserved for reasonable feature selection, as they obtained 28, 52, and 29 regional shared biomarkers, respectively. By combining the filter and embedded methods, 63 species were selected as regional shared biomarkers (Fig. 4C).

To confirm the validity of these 63 regionally shared biomarkers, we used these species as input features in RF models to distinguish between obese and healthy people across all countries. Interestingly, we found that in these countries, when the number of features reaches a certain level, the effect of classification deteriorates as the number of features increases (Fig. 4E), particularly for the UK and GER. These 63 shared regional biomarkers that contained redundant features. To address this problem, we employ sequential forward selection (a wrapper method) to reduce the redundant features. For each country, we first used the above seven embedded methods to select features and ranked them according to feature importance. Second, we increased the number of input features in the classifier model to find the optimum feature subset for each corresponding model. Third, the feature subset corresponding to the model with the best classification performance was selected as the biomarker for each country. By pooling all the countries, 52 biomarkers were obtained from the perspective of wrapper methods.

**Fig. 5:**
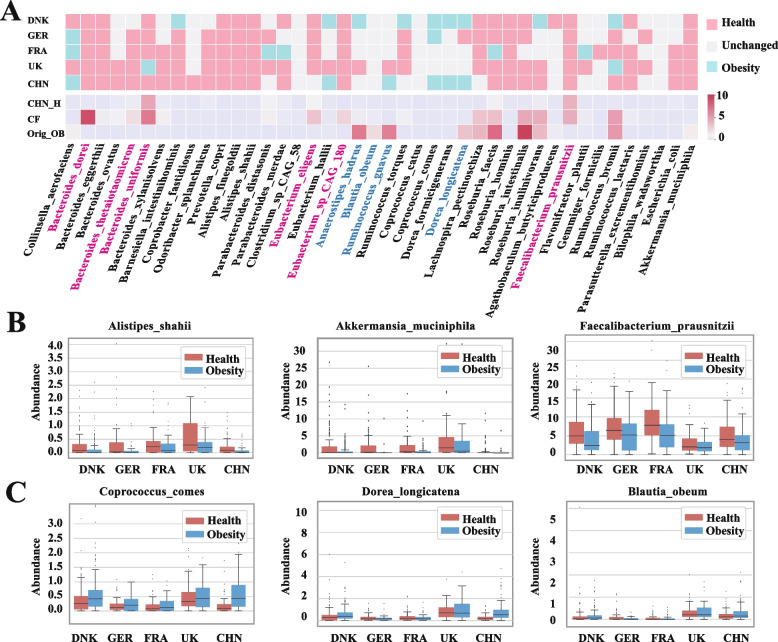
The plot shows the biomarkers identified by counterfactual inference. **A** The heatmap graph at the bottom shows the median abundance of regional shared biomarkers in the Chinese healthy individuals (CHN_H), a random Chinese original obese sample (Orig_OB), and the corresponding counterfactual instance (CF), respectively. The red-marked biomarkers mean that the abundance is increased in the counterfactual instance; the blue-marked biomarkers mean that the abundance is reduced in the counterfactual instance. The heatmap graph at the top shows the general direction of biomarkers across different countries by pooling all the counterfactual instances. The red color means that the median abundance of the biomarkers is raised in the counterfactual instances; in contrast, the blue color means that the median abundance of the biomarkers is decreased in the counterfactual instances. The gray color means that the difference in median abundance of the biomarkers between the original obese individual and the counterfactual instance is less than 0.1. **B-C** The box plots (box limits, upper and lower quartiles; center line, median; whiskers, 1.5 × interquartile range) show the abundance of biomarkers in obese and healthy people across different countries

Finally, to capture comprehensive regional shared biomarkers and remove redundant biomarkers, we took the intersection of the above two methods, with 42 species selected as final regional shared biomarkers (Fig. 5A).

### Classification performance validation of discovered regional shared biomarkers in multiple countries

To assess the validity of the selected regional shared biomarkers, we evaluated the performance of the machine learning models (seven models mentioned above) based on the selected biomarkers. Among these discovery cohorts, the results of CHN and FRA performed better than those of other countries, with the best AUC of 0.85. In contrast, the AUC of the UK only achieved 0.77 (Fig. 4F), which was the worst result among all regions. Compared with the results of the single methods for feature selection and classification, our ensemble method achieved the best classification results, indicating that our ensemble method was more effective than the others (Fig. 4F). In addition, we observed that the XGBoost model performed better than the other single models in distinguishing obese people from healthy people (mean AUC = 0.79).

Furthermore, to confirm that the selected biomarkers were not a result of overfitting in the discovery cohort, we considered additional cohorts from other countries, including 149 obese individuals and 157 healthy individuals. Using these 42 regional shared biomarkers as input features in the above ensemble models, we could also obtain a good classification effect on these additional cohorts, with a mean AUC of 0.85 (Fig. 4E). Therefore, these results verified that our regional shared biomarkers from ensemble feature selection had a significant performance for the biological datasets of the five countries considered and had good representation generalization for obesity discrimination in other countries/regions.

### Deep reinforcement learning further indicates regional common and specific modulation targets

From the above analysis, regional shared biomarkers associated with obesity have been identified; however, how to modify these biomarkers to guide obese people to become healthy remains unclear. To this end, we developed a counterfactual inference framework based on deep reinforcement learning to portray the minimal necessary changes in the biomarkers in the input space to modify the machine learning model’s predictions towards the desired target (see Methods). This is very intuitive and useful for interpreting the results of model prediction because it tells people what to do to achieve the desired outcome. Therefore, it can provide precise personalized modulation of the gut microbiome for each obese individual to help them become healthy based on the inference of machine learning models.

For example, we randomly selected one obese individual and a corresponding counterfactual instance from China. By way of counterfactual inference, we found that for this obese individual, the minimum modulation scheme was to increase the relative abundance of six bacteria (*Bacteroides dorei, Faecalibacterium prausnitzii, Bacteroides thetaiotaomicron, Bacteroides uniformis, Eubacterium eligens,* and *Eubacterium* sp. *_CAG_180*) and reduce the relative abundance of four bacteria (*Dorea longicatena, Blautia obeum, Ruminococcus gnavus, and Anaerostipes hadrus*) (Fig. 5A). In this case, an obese individual can trend towards health from the perspective of machine learning. Moreover, the relative abundances of most regulated biomarkers were closer to the median of the healthy Chinese population, which further proved the effectiveness of our method (Fig. 5A).

Despite all the shared biomarkers appearing to be associated with obesity, they may have different effects on different people in different countries. Thus, we also compared the median abundances of biomarkers for original obese individuals and counterfactual instances among various countries to determine which species are beneficial to obese people without being affected by geographical factors (regional shared). Finally, the results showed that the discovery cohorts shared several broad-spectrum taxonomic biomarkers that were altered in the same direction by counterfactual inference (Fig. 5A). Among these broad-spectrum biomarkers, *Akkermansia muciniphila*, *Faecalibacterium prausnitzii, Prevotella copri, Bacteroides dorei, Bacteroides eggerthii, Alistipes finegoldii, Alistipes shahii, Eubacterium* sp. *_CAG_180,* and *Roseburia hominis* were proven beneficial for obese individuals in these five countries (Fig. 5A). In contrast, *Coprococcus comes* and *Dorea formicigenerans* (Fig. 5A) appeared to have adverse effects on healthy individuals. To further verify the reliability of the selected broad-spectrum biomarkers, we compared the relative abundance of the biomarkers between obese and healthy individuals in each country. As a result, the direction of regulation was consistent with the abundance gap of the gut microbiome between obese and healthy individuals (Fig. 5B, C). For example, *Faecalibacterium prausnitzii* was a healthy broad-spectrum biomarker in our study (Fig. 5A), whereas the abundance of this species in healthy people was also higher than that in obese people.

### Correlation network pattern analysis on counterfactual samples

**Fig. 6:**
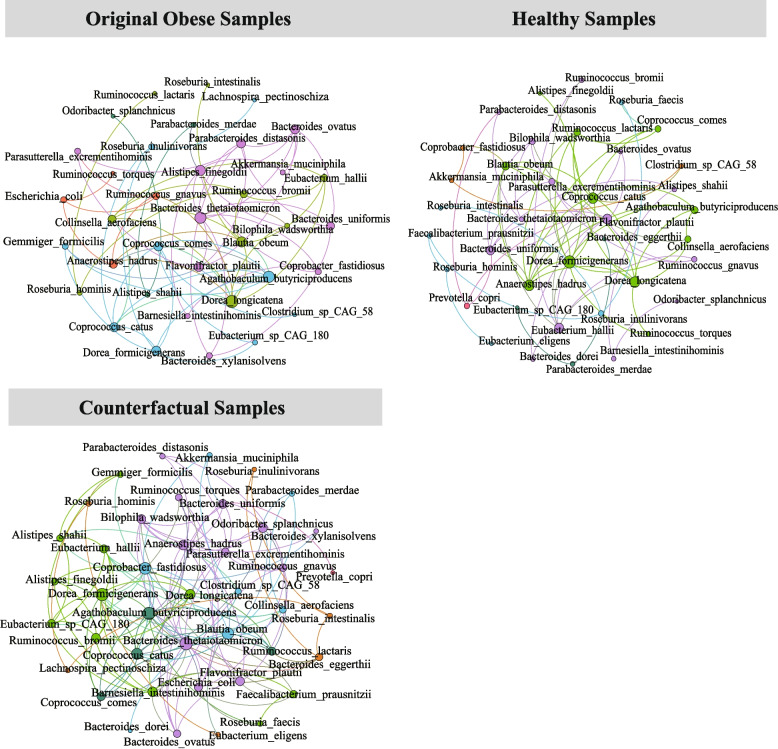
Co-abundance networks of healthy, original, and counterfactual samples. The different colors of nodes and edges indicate the species from different modules. Only the correlations of biomarkers above 0.3 are shown in this plot

To further verify the reliability of the counterfactual samples, we compared the co-abundance correlation network of healthy samples, original obese samples, and counterfactual samples based on the abundance of selected regional shared biomarkers. Notably, since the data from eastern China have a higher degree of differentiation between healthy and obese individuals, we only selected it to build and compare the co-abundance correlation network of healthy, original obese, and counterfactual samples (Fig. 6). As a result, the network of counterfactual samples is closer to that of healthy people than the original obese people. The number of common associations between the network of counterfactual samples and healthy samples (40 common associations) was greater than that between the original obese samples and healthy people (20 common associations). These recovery associations may play an important role in helping obese individuals restore their health. For example, compared to the network of original samples, the network of counterfactual samples showed four common associations with the healthy module from *Bacteroides uniformis* and four other biomarkers, suggesting that *Bacteroides uniformis* may play a key role in losing weight. In addition, we found that broad-spectrum biomarkers only have a few associations in the network, which indicates that they are not important nodes in the network. Previous studies have reached similar conclusions [26].

## Discussion

Previous studies have demonstrated that geography is a dominant factor affecting the composition of the gut microbiome. This study aimed to obtain a regional shared biomarker of obesity to decouple geographical and obesity factors and provide precise and personalized modulation analysis, thereby paving the way to make the most of the gut microbiome for obesity in the future.

First, we assumed that the most natural method for excluding the influence of geographical factors is to identify biomarkers from different countries and then take their intersection. Therefore, we present an ensemble method to obtain comprehensive biomarkers for each country based on shotgun metagenomic data from published datasets. To obtain more core biomarkers, we first eliminated biomarkers with low frequency, and 42 species were eventually selected as regionally shared biomarkers. Among these biomarkers, some have been reported to be associated with obesity in previous studies, such as *Prevotella copri* [27, 28], *Akkermansia muciniphila* [29, 30], *Faecalibacterium prausnitzii* [31, 32], and *Ruminococcus gnavus* [33, 34], indicating the reliability of our method. Compared with other feature selection methods, our method revealed more stable and effective features. For example, the GBDT feature selectors were not stable enough to obtain shared biomarkers, and the LGB, XGBoost, and XGBRF methods were not effective enough to distinguish obese people from healthy people (Fig. 4E). Among these countries, CHN had the highest AUC. To determine the reasons for this, we compared these datasets with those of other countries. We believe that the balance of these datasets plays a key role in the prediction. In contrast, there are fewer confounding factors in these datasets because the data come from studies specializing in obesity [35]. In contrast, the result of the UK was worse than that of other countries, as we missed one key biomarker. This may be attributed to the scarcity of datasets in the UK. When using the potential of these biomarkers in additional validation datasets, high-accuracy predictions were also obtained. This indicates that the selected regional shared biomarkers were not a result of overfitting in the discovery cohort. Among these machine learning models, the XGBoost classifier has been proven to be the most effective (Fig. 4E), which is in line with many previous gut microbiome studies. This suggests that we should not adhere to one classifier alone, such as an RF classifier when conducting future research on gut microbiota [36].

However, although we obtained comprehensive and precise regional shared biomarkers for obese individuals, personalized regulation of the minimum subset of the targeted gut microbiome to restore the health status of obese individuals remains a challenge. To this end, we presented counterfactual instances based on deep reinforcement learning, a strong tool to provide ideal personalized treatment regimens for obese people, which describes the necessary minimal changes in biomarkers to alter the machine learning models’ prediction of obese people to healthy. Although we are still unable to treat obese people according to the obtained precise treatment regimens because of the limitations of technology, it can still provide an option for future treatment. Furthermore, by pooling all the counterfactual explanations of obese people, we can obtain a set of beneficial broad-spectrum consistent targets for cross-regional obesity populations, such as *Akkermansia muciniphila, Faecalibacterium prausnitzii, Prevotella copri, Bacteroides dorei, Bacteroides eggerthii, Alistipes finegoldii, Alistipes shahii, Eubacterium* sp. *_CAG_180, and Roseburia hominis*. Among these biomarkers, several lines of evidence suggest the beneficial effects of *Akkermansia muciniphila, Faecalibacterium prausnitzii, Prevotella copri*, and *Bacteroides dorei* in the amelioration of obesity and associated complications [32, 34, 37]. For example, previous studies have shown that *Prevotella copri* is a potential candidate for treating metabolic diseases [38], as it can improve glucose tolerance [39], increase GLP-1 levels, and lower hunger sensations [40]. In addition, *Bacteroides eggerthii* [41]*, Roseburia hominis* [42, 43]*, Alistipes finegoldii, Alistipes shahii* [44], and *Eubacterium* sp. [45] have been shown to produce SCFAs, proving that they have the potential to help obese individuals lose weight. Conversely, *Coprococcus comes* and *Dorea formicigenerans* were positively correlated with obesity in our study, which is also consistent with previous studies [35, 46]. Certainly, these species need to be validated in further experiments. However, we also found that some shared biomarkers have poor agreement on the influence of obesity among different countries. For example, *Ruminococcus torques* plays different roles in DNK and FRA, which may be due to insufficient datasets, the same species producing different metabolites under different conditions, or the strain diversity of species [47]. It would be more precise if strain-level taxonomic datasets were used. In addition, it is worth noting that biomarkers with small changes here do not mean that they do not affect obesity but that it is easier to help obese individuals recover their health by regulating biomarkers with large changes. Furthermore, to verify the validity of the counterfactual instances, we randomly selected one counterfactual instance and the corresponding original instance for further analysis. Compared to the original obese instance, we found that most of the altered gut microbiome abundances were closer to those of healthy people. In addition, correlation network patterns were analyzed for counterfactual instances and original obese instances. It is not surprising that the counterfactual instance network has more common associations with the healthy people network than the original obese instance network. In conclusion, the counterfactual instances are closer to healthy people than the original instances in many aspects, indicating that counterfactual instances are reliable. Notably, our framework can also be applied to other diseases if we have enough relevant datasets, for instance, providing individual treatment regimens of the gut microbiome for patients with inflammatory bowel disease (IBD). However, the accuracy of the model prediction must be sufficiently high; otherwise, the results of counterfactual instances will be inaccurate.

In summary, this study focused on obtaining regional shared biomarkers by pooling shotgun metagenomic data from published datasets and providing ideal personalized treatment regimens for obese individuals. By developing ensemble methods and applying deep reinforcement learning to generate counterfactual instances, we eventually obtained and verified the regional shared biomarkers and personalized treatment regimens. However, as the geographical span of our samples was not wide enough, the obtained regional shared biomarkers are still questionable. In addition, the application of counterfactual instances in the gut microbiome is not mature, and we further need to improve the precision of the machine learning model prediction to make the results more reliable.

## Conclusions

In this study, we proposed a systematic machine-learning framework and applied it to 870 human stool metagenomes across five countries. This machine-learning framework can be divided into two parts. First, a heterogeneous ensemble feature selection diagram was established to obtain comprehensive regional shared biomarkers for obese individuals and they showed good performance in distinguishing obese people from the healthy group when demonstrated on validation datasets. Second, a deep reinforcement learning model was developed to create counterfactual instances for the gut microbiome of obese individuals. Which performed personalized counterfactual modulation analysis for individual people. The counterfactual instances were proved to be closer to healthy people than the original obese instances in many aspects, indicating that counterfactual instances are reliable. By pooling all counterfactual instances, we also inferred a set of broad-spectrum consistent targets for cross-regional obesity populations. In summary, this work helps to make the most of the gut microbiome for obesity and our machine-learning framework could also contribute to other diseases in the field of gut microbes.

## Methods

### Data collection of human stool metagenomes

We performed keyword searches (e.g., “metagenome,” “whole genome shotgun (WGS),” “gut microbiome”) in PubMed for published studies with available metagenome data of human stool. To obtain as many samples as possible from different regions, we limited ourselves to studies related to obesity and retrieved metagenomic data from studies related to other diseases, such as colorectal cancer, which had BMI (Body Mass Index) information on the samples. In these studies, wherein multiple samples were suffering from other diseases, we incorporated only samples from the control group that had not been diagnosed with other diseases and were aged > 18 years. Raw metagenome sequence files were downloaded from the National Center for Biotechnology Information and European Nucleotide Archive databases. Furthermore, all samples were categorized by country and BMI (obesity group: BMI ≥ 30; healthy group: BMI < 25) to facilitate further study.

### Bioinformatics processing

In this study, Trimmomatic (SLIDINGWINDOW:4:25 MINLEN:60 LEADING:3 TRAILING:3) was used to remove low-quality read bases. Thereafter, the remaining reads were compared with the human genome using the BWA tool to remove reads from the host. Finally, the composition of the microbiome was determined using MetaPhlAn2 [48] software by inputting quality-controlled short-read sequences, and the species abundance information tables of the individual samples were combined using merge_metaphlan_tables.py scripts.

### Calculation of microbiome diversity

We used the R package “vegan” v2.5.6 to calculate the Shannon diversity index based on the species abundance of our samples. To explore the differences in the Shannon index between different groups, the Wilcoxon rank-sum test was used in the basic R package. At the same time, the R packages “vegan” v2.5.6 and “ade4” v1.7–15 were used to perform PCoA ordination with Bray-Curtis distance computed on the microbiome taxonomic profiles.

### Statistical analysis

There are various approaches to calculating the association between the gut microbiome and BMI with varying efficiency and accuracy. To obtain more comprehensive and accurate biomarkers for obesity, we selected seven filter correlation algorithms. As a simple popular indicator, Pearson’s correlation coefficient was considered. In addition, Spearman’s correlation coefficient, Kendall’s correlation, and maximal information coefficient (MIC) were used to find nonlinear associations. Finally, Pearson’s, Spearman’s, and Kendall’s partial correlation coefficients were included to detect direct interactions between them.

Pearson’s, Spearman’s, and Kendall’s correlation coefficients were calculated using Python v3.8.5. As mentioned above, we also obtained MICs using the Python package “minepy”. In contrast, Pearson’s, Spearman’s, and Kendall’s partial correlation coefficients were determined from the Python package “Pingouin” with method = “Pearson,” “Spearman,” and “Kendall,” respectively.

### Machine learning

In this study, machine learning models were employed to select features and evaluate their quality. Specifically, we used Decision Tree (DT), Random Forest (RF), Gradient boosted regression trees (GBDT), eXtreme Gradient Boosting (XGBoost, XGBRF), Adaptive Boosting (AdaBoost), and LGB (Light Gradient Boosting Machine (LGB) to select the features through embedding in the modeling phase based on the scikit-learn and XGBoost Python packages, which have been widely applied in the field of the gut microbiome [49]. To verify the accuracy of the selected features, we used them as the features of the training models and evaluated the AUC of these models with 5-fold cross-validation.

### Counterfactual inference for modulation analysis

In this study, counterfactual reasoning was constructed to alter the targeted microbiome biomarkers so that the regulated features were close to the expected instance (using distance measure) and could not be distinguished from real instances. In summary, we built a classification model that can accurately distinguish obesity from healthy samples based on the abundance of shared biomarkers. Second, we attempted to alter the composition of obese samples to their healthy counterparts by modulating the abundance of biomarkers (construct counterfactual explanations). Thus, these counterfactual explanations can provide personalized modulation analysis for each individual with obesity. The method to obtain counterfactual explanations of the original obese individuals can be defined as:

1 $${y}_M=M(x)$$

2 $${x}_{CF}=x+{\delta}_{CF}$$

3 $${y}_T=M\left({x}_{CF}\right)$$

Let *M* be a classifier model, *x* an original obese instance, *y*_*M*_ the model prediction of *x*, *x*_*CF*_ a counterfactual instance, *δ*_*CF*_ the changes in features, and *y*_*T*_ the target prediction. For each original obese instance, we needed to produce a counterfactual instance *x*_*CF*_ (Eq. 2) that altered the model prediction to health (Eq. 3) by changing the abundance of biomarkers *δ*_*CF*_ in this work. To obtain a suitable *x*_*CF*_, we applied deep reinforcement learning to get *δ*_*CF*_ of each original obesity instance. Compared to other methods, it is more effective and suitable for high-dimensional datasets. To better understand the advantages of our method, we provide a simple review of reinforcement learning here. In addition, the nature of reinforcement learning is that an agent tries to obtain the maximum cumulative reward by repeatedly interacting with the environment. One of the classic approaches to obtaining the best policy in reinforcement learning is to approximate the Q-function, which is an effective function for estimating the reward value after the agent takes a particular action in a given state. Therefore, if we know all the results of any action in a state *s* by the action-value function *Q*^∗^(*s*, *a*), we will find the best strategy to acquire the maximum cumulative reward by

4 $${a}^{\ast }(s)= \mathop{argmax}\limits_{a}\ {Q}^{\ast}\left(s,a\right)$$

We can easily obtain *Q*^∗^(*s*, *a*) by evaluating the state-action pair for all available actions in a discrete action space. However, it is usually impossible to determine the optimal *Q* function for a continuous action space. To this end, Deep Deterministic Policy Gradient (DDPG) [50] was proposed by interleaving a state-action function approximator *Q* (the critic) of *Q*^∗^(*s*, *a*) with learning an approximator 𝜇 (the actor) for the best action *a*^∗^(*s*), which optimized the parameters through gradient-based methods.

Returning to this theme, because the abundance of biomarker *δ*_*CF*_ is a continuous vector, we applied DDPG to find the best *δ*_*CF*_ in this study [51]. Specifically, the DDPG includes two separate networks, an actor 𝜇 (take action by generating *δ*_*CF*_) and a critic *Q* (evaluate the effectiveness of *δ*_*CF*_). The training steps aim to optimize the actor-network and the critic network. For the actor-network, it is designed to maximize the critic *Q* output *L*_*max*_ (Eq. 5) by generating the *x*_*CF*_. Rather than straightforwardly put the *x* in the high-dimensional information space to generate the *x*_*CF*_, we first trained an autoencoder model. The encoding of *x* can be defined as *z* = *enc* (*x*), and the actor generates the counterfactual instance *z*_*CF*_ = *μ*(*z*, *y*_*M*_, *y*_*T*_, *c*; *θ*_*μ*_), 𝑐 represents the conditioning vector, *θ*_*μ*_ is an important parameter for *μ*. For the subsequent analysis of the *x*_*CF*_, the *z*_*CF*_ will be decoded back to the original instance as *x*_*CF*_ = *dec* (*z*_*CF*_). In addition, the actor should also minimize *x* and *x*_*CF*_ to generate a sparse counterfactual explanation. Therefore, the sparsity loss *L*_*sparsity*_ (Eq. 6) was generated to operate on the *x*_*CF*_ and combine the *L*_1_ loss over the features. At last, the consistency loss *L*_*consist*_ (Eq. 7) was employed to encode the *x*_*CF*_ back to the *z*_*CF*_. The *L*_*max*_, *L*_*sparsity*_, and *L*_*consist*_ can be separately defined as follows:

5 $${L}_{max}=-\frac{1}{\left|B\right|}\sum_BQ\left(z,{y}_M,{y}_T,c,{z}_{CF}\right)$$

6 $${L}_{sparsity}=\frac{1}{\left|B\right|}\sum_B{L}_1\left(x,{x}_{CF}\right)$$

7 $${L}_{consist}=\frac{1}{\left|B\right|}\sum_B{\left( enc\left({x}_{CF},c\right)-{z}_{CF}\right)}^2$$

And we can update the actor by one-step gradient descent using:

8 $${\nabla}_{\theta_{\mu }}\left({L}_{max}+{\lambda}_s{L}_{sparsity}+{\lambda}_c{L}_{consist}\right)$$

Here, *B* is a batch of experiences, *z*_*CF*_ = *μ*(*z*, *y*_*M*_, *y*_*T*_, *c*; *θ*_*μ*_), *θ*_*μ*_ is an important parameter for the *μ*, and *λ*_*s*_ and *λ*_*c*_ are the loss hyperparameters that determine the effects of *L*_*sparsity*_ and *L*_*consist*_, respectively. We updated the *θ*_*μ*_ parameter through gradient descent to find the best *z*_*CF*_ by the actor 𝜇 so that maximize the critic *Q* (output)

The critic network regresses on the reword value *R* = *f*(*M*(*x*_*CF*_), *y*_*T*_), which is determined by the prediction of the machine learning model. To make critic *Q* more accurate, the DDPG update critic by one-step gradient descent can be defined as:

9 $${\nabla}_{\theta_Q}\frac{1}{\left|B\right|}\sum_B\left(Q{\left(z,{y}_M,{y}_T,{z}_{CF},c;{\theta}_Q)-R\right)}^2\right)$$

Similarly, *θ*_*Q*_ is an important parameter for critic *Q*. We updated the *θ*_*Q*_ parameter by gradient descent so that the critic *Q* could be closer to the *R.* Therefore, the critic *Q* can be more reliable to instruct the actor 𝜇 to obtain the optimal *x*_*CF*_.

### Co-abundance networks of original obese, healthy, and counterfactual samples

To further verify the reliability of the counterfactual samples, we compared the co-abundance correlation network of healthy samples, original obese samples, and counterfactual samples based on the abundance of selected regional shared biomarkers. Correlations between biomarkers were computed using Pearson’s correlation coefficient in the Python environment v3.8.5. Gephi v0.9.2 was employed to visualize the co-abundance network.

## Data Availability

Raw human stool metagenomic data were obtained using the European Nucleotide Archive (https://www.ebi.ac.uk/ena/browser/home) identifiers PRJEB12123, PRJEB9576, PRJNA539850, PRJEB37249, PRJNA665061, PRJEB4336, PRJEB1786, PRJEB1220, and PRJEB7774.
